# A Radiomics-Based Machine Learning Perspective on the Parotid Gland as a Potential Surrogate Marker for HPV in Oropharyngeal Cancer

**DOI:** 10.3390/cancers15225425

**Published:** 2023-11-15

**Authors:** Gordian Prasse, Agnes Glaas, Hans-Jonas Meyer, Veit Zebralla, Andreas Dietz, Kathrin Hering, Thomas Kuhnt, Timm Denecke

**Affiliations:** 1 Department of Radiology, University of Leipzig, 04103 Leipzig, Germany; hans-jonas.meyer@medizin.uni-leipzig.de (H.-J.M.); timm.denecke@medizin.uni-leipzig.de (T.D.); 2 Department of Radiation Therapy, University of Leipzig, 04103 Leipzig, Germanythomas.kuhnt@medizin.uni-leipzig.de (T.K.); 3 Department of Otorhinolaryngology, Head and Neck Surgery, University of Leipzig, 04103 Leipzig, Germany

**Keywords:** OPSCC, radiomics, HPV, parotid gland, machine learning

## Abstract

**Simple Summary:**

The human papillomavirus (HPV) is an important prognostic marker in oropharyngeal squamous cell carcinoma (OPSCC) due to its involvement in carcinogenesis. The presence of the virus itself or markers of a past infection are usually determined by tissue specimen. This work applies predictive machine learning models to objectified imaging features extracted from routine imaging of patients newly diagnosed with OPSCC to classify cases of HPV-positive and negative tumors. In addition to results and classification performance being in line with existing literature on imaging-based HPV classification, imaging features of the parotid gland could be shown to yield relevant information. Parotid gland imaging offers potential as a screening tool and may aid clinical decision making in cases of cancer with unknown primary in the future.

**Abstract:**

Background: In treatment of oropharyngeal squamous cell carcinoma (OPSCC), human papillomavirus status (HPV) plays a crucial role. The HPV-positive subtype tends to affect younger patients and is associated with a more favorable prognosis. HPV-associated lesions have been described in the parotid gland, which is included in routine imaging for OPSCC. This work aims to explore the ability of an ML system to classify HPV status based on imaging of the parotid gland, which is routinely depicted on staging imaging. Methods: Using a radiomics approach, we investigate the ability of five contemporary machine learning (ML) models to distinguish between HPV-positive and HPV-negative OPSCC based on non-contrast computed tomography (CT) data of tumor volume (TM), locoregional lymph node metastasis (LNM), and the parotid gland (Parotid). After exclusion of cases affected by streak artefacts, 53 patients (training set: 39; evaluation set: 14) were retrospectively evaluated. Classification performances were tested for significance against random optimistic results. Results: The best results are AUC 0.71 by XGBoost (XGB) for TM, AUC 0.82 by multi-layer perceptron (MLP) for LNM, AUC 0.76 by random forest (RF) for Parotid, and AUC 0.86 by XGB for a combination of all three regions of interest (ROIs). Conclusions: The results suggest involvement of the parotid gland in HPV infections of the oropharyngeal region. While the role of HPV in parotid lesions is under active discussion, the migration of the virus from the oral cavity to the parotid gland seems plausible. The imaging of the parotid gland offers the benefit of fewer streak artifacts due to teeth and dental implants and the potential to screen for HPV in cases of an absent or unlocatable tumor. Future investigation can be directed to validation of the results in independent datasets and to the potential of improvement of current classification models by addition of information based on the parotid gland.

## 1. Introduction

While exposure to alcohol and tobacco is historically considered a risk factor for the development of oropharyngeal squamous cell carcinoma (OPSCC), infection with human papillomavirus (HPV) is among the factors generally considered in therapeutic concepts [1,2,3,4]. Current data suggest the subclassification of OPSCC cases into two phenotypes regarding their distinct clinical and biological features: individuals with a history of smoking and drinking habit showing no HPV infection and occurrences in younger patients, with 40 years of age considered as a threshold, without a history of smoking and drinking but associated with HPV infection. HPV-positive OPSCC is associated with a more favorable prognosis [1,5,6,7].

While there are reports on diverging imaging features of both primary tumor site and affected lymph nodes depictable on computed tomography (CT) and magnetic resonance imaging (MRI), differentiation of HPV infection status by cross-sectional imaging on an individual basis is still considered challenging [8]. The radiomics research field leverages reproducible quantitative representations of gray value patterns to convert medical imaging data into minable high-dimensional feature space to facilitate machine-based analysis [9,10,11]. In head and neck squamous cell carcinoma (HNSCC), the radiomics approach based on CT has successfully been used for both risk stratification and HPV status prediction [12].

Since the presence of high-risk HPV in parotid lesions has been reported, there is an ongoing debate on the significance of published findings and the contribution of HPV infection to pathogenesis in parotid malignancies [13,14]. We aim to investigate the radiomics approach’s ability to classify HPV status based on imaging of the parotid gland in OPSCC.

## 2. Materials and Methods

### 2.1. Patient Cohort

This study was approved by the ethics committee of the University Hospital of Leipzig (ethics approval number 030/19-ek). Patients who underwent OPSCC radiochemotherapy at the Department of Radiooncology of the University Hospital Leipzig between November 2014 and September 2020 were screened for inclusion criteria. Inclusion criteria were (1) histopathologically confirmed OPSCC, with documented HPV status; (2) no previous therapy; and (3) non-contrast CT imaging of the neck dated before therapy initiation. The presence of HPV was tested in clinical routine using immunohistochemistry of the p16 protein [15].

### 2.2. Imaging and Feature Extraction

Non-contrast CT scans acquired before therapy and used for radiation planning were identified in the local picture archive and communication system (PACS). The presence of streak artifacts was rated by an experienced radiologist on a semiquantitative scale from 0 to 2: no relevant artifacts (0); minor artifacts affecting local tumor mass, lymph node metastases, or parotid glands on two consecutive slices at most (1); and significant artifacts affecting analyzed structures (2). Cases with an artifact score of 2 were excluded. Contouring of the gross tumor volume (GTV) of the primary tumor (TM), the lymph node metastases (LNM), and the parotid gland (Parotid) was performed by an experienced radiooncologist and an experienced radiologist in consensus, both blinded to HPV status. Since the imaging was performed on one of three scanners (Biograph PET/CT, ‘No. 1’; Somatom Confidence, ‘No. 2’; Somatom Emotion, ‘No. 3’; all Siemens, Erlangen, Germany), the scanner and the convolution kernel used for image reconstruction were introduced as confounder variables.

Pyradiomics (Version 3.0.1) [16] was used to extract first-order features, shape features, and gray level co-occurrence matrices (GLCM) texture features of all contours using the same settings (removeOutliers: 3, resegmentRange: −200–350, resegmentMode: ‘absolute’, resegmentShape: False, interpolator: ‘sitkBSpline’, resampledPixelSpacing: [1, 1, 3], binWidth: 8).

### 2.3. Machine Learning Methods

Five popular ML models were applied to avoid bias towards a single model or model type: logistic regression (LR), multi-layer perceptron (MLP), random forest classifier (RF), linear support vector classifier (SVC) [17], and extreme gradient boosting classifier (XGB) [18,19].

LR is a widely used and comparatively simple method of classification. Unlike linear regression, which models a continuous variable, LR models the probability of a class membership by transforming multiple input variables into one dependent variable into the range between 0 and 1, reflecting a probability. In the optimization process, variables are removed and added step-wise to improve the prediction.

MLP provides a series of linear or non-linear transformations organized in nodes and layers, originally inspired by the architecture of neurons in the human brain [20]. Via iterative optimization of the transformation parameters, multivariate information can be passed through the network layers and condensed into probabilities of class membership [21].

RF is an ensemble model that consists of multiple decision trees, each fitted to a random subset of the input data. The probability of class membership is determined by the majority vote in the ensemble [22].

SVC is a model that aims to separate classes in the multidimensional space formed by the predictor variables by a multidimensional plane within that space. The model fitting process includes optimization of both the plane curvature and maximizing the distance between the nearest data points of each class [23].

XGB, like RF, is a tree ensemble model [24]. In addition to fitting the tree ensemble to the predictor variables, XGB iteratively introduces new regression trees to compensate for errors made previously. This work uses the popular implementation XGBoost [19].

### 2.4. Dataset Creation and Model Evaluation

Nine datasets were created on a per-lesion basis, consisting of first-order features, shape features, and GLCM texture features for the segmentations of TM, LKM, and Parotid. To evaluate metrics of individual lesions or parotids respectively against a combination of metrics of all three (TM, LKM, Parotid), three further datasets were created using a combinatory approach: all possible combinations per patient of each one of TM, LKM, and Parotid were taken as data points. Several data points per patient were possible. Not all targets were segmented in all cases.

To optimize the radiomics dataset for reproducibility and generalizability, feature selection using Shapley values and case selection using Data Shapley were performed. Based on game theory, Shapley values can be assigned to features, reflecting their influence on the decision of a classifier [25,26,27]. In the selection process, features were sorted by descending absolute mean Shapley value, and the top-ranking features were selected, limited to 10% of the number of cases per dataset, as previously suggested [28]. Similarly, based on game theory concepts, individual data points can be assigned values according to their marginal contribution to the classifier training. The Data Shapley method was proposed to optimize training data to filter misclassified or noisy data during training and improve generalization [29,30].

The evaluation was performed on a hold-out test dataset, which was selected by sorting the cases by time of diagnosis and taking the most recent cases until at least 25% of all cases were reached, and an even distribution of HPV-positive and negative cases was achieved. The remaining cases were assigned to the training dataset. This way, the hold-out evaluation set was optimized to avoid bias in evaluation metrics [31], and data acquisition of the groups was temporarily distinct to facilitate generalization evaluation. There was no case in both the training and test dataset to avoid information leakage. To determine the likelihood of the score occurring randomly, 1000 train-and-validation runs on the training datasets with randomized labels and three-fold cross-validation were conducted, and the *p*-value was estimated by the proportion of scores above the mean evaluation score using unaltered labels [32]. In the final step, the area under the receiving operator curve (AUC) was used as evaluation metric on the hold-out test set. Mean AUC values and 95% confidence intervals were determined by 100 Monte Carlo sampling simulations.

## 3. Results

### 3.1. Patients and Datasets

**Figure 1:**
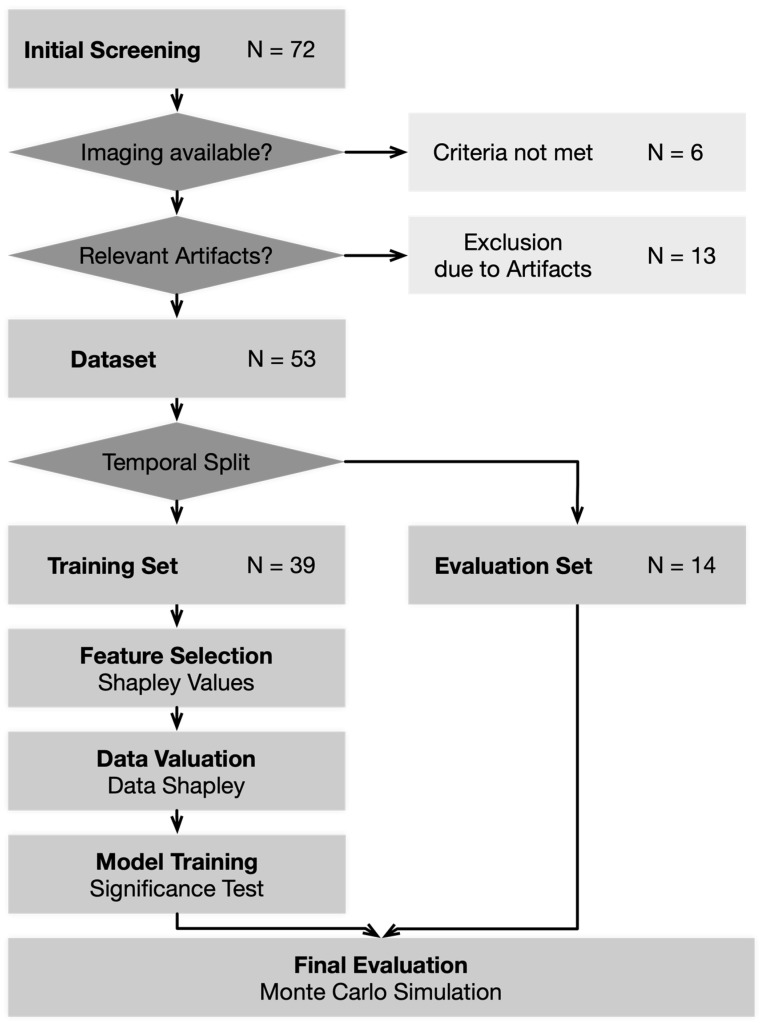
Schematic illustration of the analysis.

Seventy-two cases were identified in the initial screening. For six cases, there was no non-contrast CT scan available before therapy initiation. Thirteen cases were excluded due to severe artifacts. A schematic illustration of the analysis is provided in Figure 1. Table 1 gives an overview of the group compositions, Table S2 shows the patient characteristics by HPV status.

### 3.2. ML Model Performances

**Figure 2:**
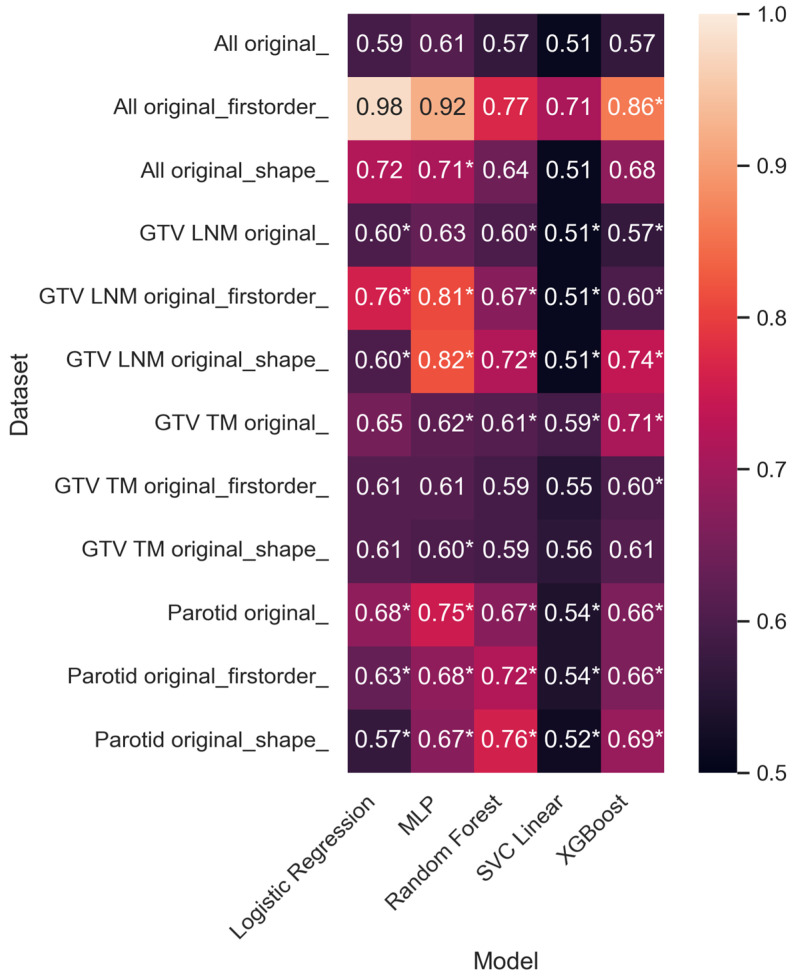
Heatmap of ML model performance per dataset based on area under the receiving operator curve (AUC). Given are mean AUC values of 100 Monte Carlo (MC) simulations in which models are trained on randomly chosen data points of the given datasets and evaluated on the same corresponding test set that consists of 26% hold-out test cases. Test cases are never used during training. A *p*-value was estimated by comparison to 1000 training-and-evaluation runs on the training set with randomized labels to quantify the probability of the performances occurring randomly. *p*-Values < 0.05 are marked with an asterisk (*).

**Figure 3:**
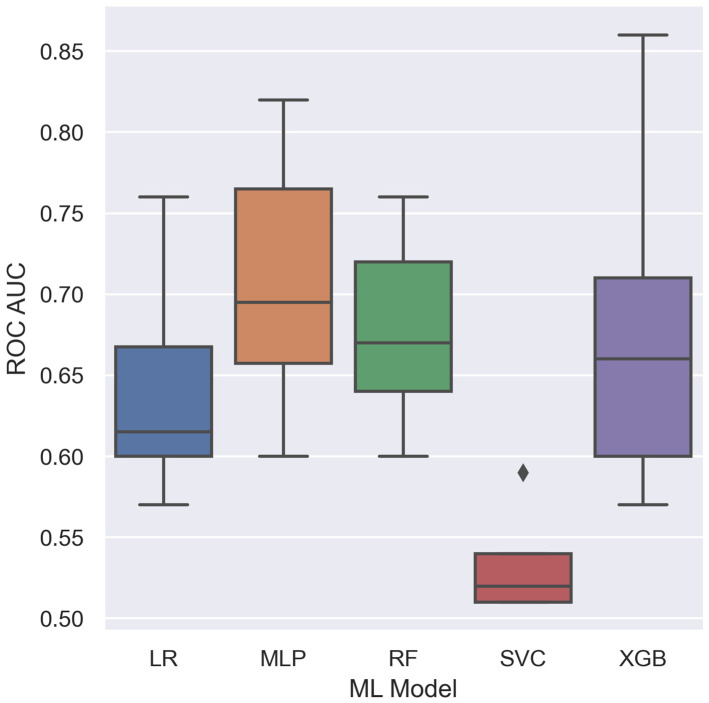
Boxplot of model evaluation performances on the hold-out test set by ROC AUC.

The training model fit showed significant results in 36 of 60 dataset/ML model combinations (60%). XGB reached the highest significant AUC of 0.86 in the All original_firstorder_ dataset. Per dataset, the highest results were 0.82, 0.71, and 0.76 for GTV LNM, GTV TM, and Parotid, respectively. Figure 2 summarizes the AUC results. Table 2 provides an overview of the AUC values and the corresponding *p*-values. Sample size varied between the different datasets due to the lesion-wise approach and selection of data points by data valuation; Table 3 provides the corresponding overview. For details of the feature selection results, the reader is referred to Table S1. As measured by significant AUC values, MLP shows the highest mean value, SVC the lowest, and XGB the highest range (see Figure 3).

## 4. Discussion

This work used imaging data of primary tumor masses (GTV TM), lymph node metastases (GTV LNM), and parotid glands to analyze their ability to distinguish between HPV-positive and negative cases in OPSCC. It could be shown that machine learning models can condense meaningful information from radiomics data derived from non-contrast CT scans. The best performance was observed for the dataset that was synthesized of the features of one GTV TM, one GTV LNM, and one Parotid gland per case; the XGB model reached an AUC of 0.86 on the validation dataset (0.36, 0.88, 0.90, and 0.43 for sensitivity, specificity, PPV, and NPV, respectively). Summarizing information from more than one ROI type poses the challenge of including more than one ROI per case. This lesion-wise analysis addressed this by creating a synthetic dataset composed of combinations of the different ROI types per case without repetitions. To contribute to the dataset, a case has to include at least one of each ROI type, hence the low sample size (n = 29). The largest sample size is to be found in the LNM dataset (n = 89). In this set, the MLP classifier scored an AUC of 0.82 (0.11, 0.90, 0.78, and 0.41 for sensitivity, specificity, PPV, and NPV, respectively), remarkably only using shape features. In the Parotid shape dataset, the RF classifier reached the top score of AUC 0.76 (0.10, 0.91, 0.47, and 0.51 for sensitivity, specificity, PPV, and NPV, respectively). Using all available features, the MLP classifier scored AUC 0.75 (0.32, 0.87, 0.73, and 0.58 for sensitivity, specificity, PPV, and NPV, respectively). On the GTV TM dataset, the XBG classifier scored AUC 0.71 (0.43, 0.86, 0.75, and 0.71 for sensitivity, specificity, PPV, and NPV, respectively).

**Figure 4:**
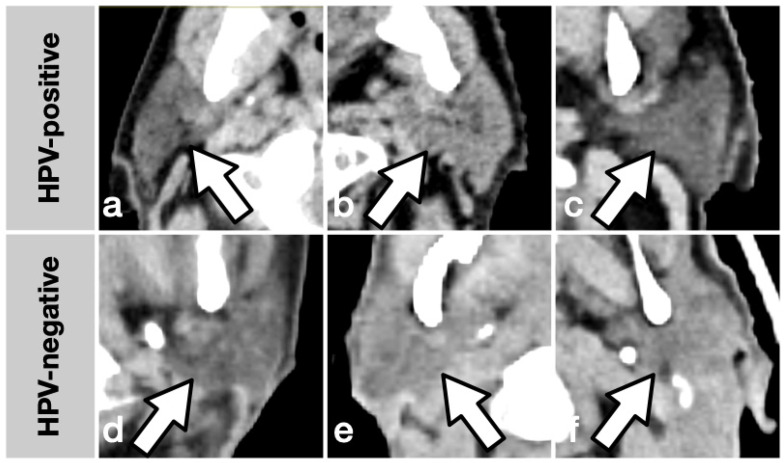
Illustrative imaging samples of HPV-positive (top row, (**a**–**c**)) and negative (bottom row, (**d**–**f**)) cases. (**a**–**c**) show parotid glands that score low sphericity (0.56; 0.57; 0.58) and high interquartile range (44; 41; 36), and (**d**–**f**) show parotid glands that score high sphericity (0.65; 0.66; 0.67) and low interquartile range (21; 23; 23), in relation to the whole sample.

Selecting an appropriate set of features from a dataset poses a non-trivial challenge when the number of features exceeds the sample size. While a wide range of algorithms is proposed for feature selection and ready-to-use implementations are publicly available, no generally accepted optimal solution fits every scenario, and feature selection remains a potential statistical pitfall [28]. For this to work to yield explainable results utilizing a machine learning approach, in addition to the whole number of features (original_, n = 107), features describing the distribution of Hounsfield units within an ROI (original_firstorder_, n = 20) and features describing the ROI shape (original_shape_, n = 16) were meaningfully grouped and analyzed separately. Feature selection was performed utilizing SHAP, a novel framework based on game theory that unifies six established methods and allows model-agnostic measurement of feature importance [26,27]. In the best-performing dataset, two features were selected as most important: GTV_LNM__original_firstorder_Minimum and PAROTID__original_firstorder_RootMeanSquared. Indeed, this choice represents features expected to be relatively resilient to imaging artifacts or variance in ROI precision taken from two different ROI types. In the LNM_original_shape dataset, one confounder variable was selected as relevant: the convolution kernel. Among the features selected in the Parotid original_ dataset is PAROTID__original_firstorder_RootMeanSquared, which is also found in the mentioned best-performing All original_firstorder_ dataset. GTV_LNM__original_firstorder_Minimum, the other feature, was selected in both the GTV LNM original_firstorder_ and the GTV LNM original_shape_ dataset. In classification tasks using radiomics methodology, a relevant number of features are usually strongly correlated with each other. Multivariate ML models are capable of handling a large number of features, but redundant, noisy information will likely lead to inconsistent results. In the presence of strong correlations among the features, model-based selection methods are prone to inconsistent selections since correlated features are interchangeable, possibly impeding inference of causal relationships. The fact that the best-performing datasets share common most important features gives strong support for SHAP as an adequate selection tool for the current task. Figure 4 gives six samples of the characteristics of parotid glands of the study population, illustrating the possible use of two arbitrary features that can be potentially visually assessed: sphericity objectifies a shape’s divergence from a perfect sphere where values closer to zero indicate a more complex surface, and the interquartile range of Hounsfield units (HU) is a measure of inhomogeneity with higher values indicating more contrast in voxel values.

The involvement of HPV in the development of parotid gland lesions is subject to active discussion in public research. In 2007, Vageli et al. [13] described the presence of high-risk HPV genotypes in nine parotid lesions, mixed benign and malignant. In 98 analyzed mucoepidermoid carcinomas of the parotid gland, a 2013 study found transcriptionally active HPV-16 (in 30%), HPV-18 (in 13%), or both (in 7%) [33]. A 2014 study found an HPV-positive rate of 59.6% in parotid gland benign tumor tissues and 42.9% in parotid malignant tissues in 59 tumors, dominated by high-risk subtypes [34]. The concomitantly analyzed group of 20 cases of normal oral mucosa was HPV-negative. Descamps et al. [35] reported a low prevalence in 79 benign and malignant parotid lesions. In a 2014 study, Bishop et al. [36] did not find one HPV-positive lesion in 43 mucoepidermoid carcinomas of the parotid gland. The Stenson’s duct that connects the parotid gland to the oral cavity is a possible route for HPV migration. Given the well-documented role of HPV in OPSCC, its involvement in carcinogenesis in the parotid gland seems plausible. The issue remains under active discussion since several studies failed to reproduce results, and aside from reported prevalence, evidence for a causal role remains to be found [14]. While, to the authors’ knowledge, there are no data on HPV prevalence in healthy individuals’ parotid glands, this work proves that an HPV infection of the oropharyngeal region, past or active, leaves a distinct pattern in non-contrast CT.

The HPV status challenge initiated by MICCAI/M.D. Anderson Cancer Center Head and Neck Quantitative Imaging Working Group used ROIs of GTV TM and GTV LNM on contrast-enhanced CT imaging of the head and neck region to classify HPV status [37]. The final evaluation was performed on a private hold-out dataset inaccessible to the competing teams. The winning model of 2016 was LR based on only two features: the mean width of the ROI and spherical disproportion, an equivalent to sphericity in this work; it scored AUCs of 0.75 and 0.91 on the training set and the final evaluation, respectively [38]. In contrast to this work, the analysis was performed case-wise, and in cases with more than one ROI, one synthetic ‘consensus ROI’ was created by taking the most extreme value per feature per case. Also, all ROIs per case were considered the same, neglecting potential biological differences between GTV TM and GTV LNM. Feature selection was carried out using statistical means in a staged process by excluding features that were unstable within the training group, showed equal distribution between the classes, or had a low correlation coefficient. In this work, LR also scored the highest AUC using only two features, but without statistical significance, this result must be interpreted with caution.

The different phenotypes of HPV-positive and negative OPSCC led to the concept of therapy de-escalation in HPV-positive cases in order to limit the side effects of radiochemotherapy. While there is an ongoing debate on the details of de-escalation and patient selection, current efforts go towards individualized therapy concepts [39]. The evaluation of HPV status by medical imaging offers the future possibility of rendering solid tissue sampling for immunohistochemistry unnecessary and reducing costs [40]. In the US, together with the prevalence of HPV infections, the incidence of OPSCC has been increasing in the past two decades [3,41]. Gaining information from the parotid glands in a non-invasive way opens the possibility of being beneficial in the following clinical scenarios: absence of cancer tissue that is accessible to sampling, cancer with unknown primary (CUP), contraindications to or missing patient consent for tissue sampling, and screening for individuals at risk prior to a cancer diagnosis.

There are limitations to the interpretability of this study’s results. First, the small sample size, especially in the synthetic All dataset, limits the ability of the trained ML models to generalize. While a higher sample size can be expected to positively affect both the generalization gap on unseen test data and classification performance, the current data are sufficient to state that ML models can condense meaningful information from TM, LNM, and parotid glands, with statistical significance that was estimated by permutations [32]. Validation was performed on data acquired at one institution. After careful consideration, non-contrast CT was selected as the imaging modality of choice due to the mixed availability and inconsistent protocols of more advanced imaging, such as contrast-enhanced CT or MRI, in this retrospective cohort. Still, there is relevant heterogeneity regarding the scanners and reconstruction kernels used; one of the three scanners is not used in the test set. Further obstacles are introduced by use of low-dose attenuation-correction CT of PET/CT imaging. A temporal split of the cases ensures that there is no information leakage between training and test set. Second, the model performance is also limited by the nature of the non-contrast radiation planning scans. In other tasks, this modality yields no additive information, e.g., prediction of pathological complete response to radiochemotherapy in rectal cancer [31]; or scores lower in classification tasks compared to contrast-enhanced CT, e.g., in differentiation of sacral chordomas from sacral giant cell tumors [42]. Public challenges for classifying HPV status from CT imaging not only deliver a proof of concept but also show remarkable generalizability [37,38]. A higher sample size will likely improve HPV classification by parotid gland, opening the possibility for screening for individuals at risk. Third, streak artefacts and variation in precision of the ROIs likely affect classification performance and the ability to select the most meaningful features or draw conclusions from the analysis. The influence of extreme HU values due to streak artifacts on the radiomics data is limited by preprocessing, as voxels outside the range of −200–350 HU were excluded from further calculations. Furthermore, all imaging was visually assessed, and cases with relevant artifacts were excluded. Still, streak artifacts are frequent in head and neck CT imaging. They cannot be removed entirely by postprocessing, potentially facilitating the use of shape and size features that are, by nature, independent of the voxel values. Fourth, the delineation of the ROIs depends on expert knowledge and is subject to imaging quality and artifacts. GTV TM of OPSCC is usually found in closer proximity to, if not on the same slice as, streak artifacts caused by teeth and dental implants and is, thus, more likely to yield less stable, reliable objectified imaging features in CT compared to GTV LNM or the parotid glands. Furthermore, precise ROI placement depends on the anatomical structures of interest to have clearly identifiable borders, a property that is impaired in the presence of infiltrative carcinoma tissue. Fifth, this work retrospectively analyses data, opening the possibility of investigator bias. To cope with bias, the ROI-placing physicians were blinded to HPV status. The presence of HPV is determined by p16 immunohistochemistry. There is broad evidence that p16 over-expression is very sensitive to detecting transcriptionally active HPV; its correlation with overall survival and outcome in OPSCC is well documented in the literature [15]. The use of p16 immunohistochemistry as a standalone test is recommended in clinical practice and for clinical studies [43,44].

## 5. Conclusions

Classification performances of HPV status based on non-contrast CT imaging of the parotid gland and TM and LNM are comparable, suggesting involvement of the parotid in HPV infections of the oropharyngeal region. While the sample size of this work is insufficient to robustly estimate the AUC for potential use on external data due to the generalization gap, popular ML models show the ability to statistically significantly condense meaningful information from radiomics features of the parotid gland. Using both the parotid as ROI and shape features as model input contributes to more reproducible results than TM and LNM, which are more commonly affected by streak artefacts. Potential clinical benefits may arise in the use cases of CUP and screening for individuals at risk. The potential accuracy of methods based on the parotid needs to be evaluated in future studies utilizing a larger sample size.

## Figures and Tables

**Table 1:** Descriptive statistics of the patient cohort.

	All	Train	Test
**Patients [*n*]**	53	39	14
**Mean age [years] (range)**	60.8 (41–91)	60.1 (45–80)	62.6 (41–91)
**Male [%]**	90.6	94.9	78.6
**HPV positive [%]**	37.7	33.3	50.0
**T stage**			
** T4**	35	27	8
** T3**	9	5	4
** T2**	5	4	1
** T1**	4	3	1
**N stage**			
** N2**	38	28	10
** N3**	5	4	1
** N1**	5	3	2
** N0**	5	4	1
**M stage**			
** M0**	49	36	13
** M1**	3	2	1
**Grading**			
** G2**	31	21	10
** G3**	21	17	4
** G1**	1	1	0
**Scanners**			
** No. 1**	20	14	6
** No. 2**	19	11	8
** No. 3**	14	14	0

**Table 2:** Performance evaluation results. *p*-Values < 0.05 are marked with an asterisk (*).

ML Model	LR		MLP		RF		SVC		XGB	
Dataset	AUC	*p*-Value	AUC	*p*-Value	AUC	*p*-Value	AUC	*p*-Value	AUC	*p*-Value
All original_	0.59 [0.50–0.75]	0.066	0.61 [0.50–0.75]	0.102	0.57 [0.50–0.75]	0.185	0.51 [0.50–0.62]	0.092	0.57 [0.50–0.69]	0.101
All original_firstorder_	0.98 [0.75–1.00]	0.168	0.92 [0.56–1.00]	0.129	0.77 [0.50–1.00]	0.137	0.71 [0.50–1.00]	0.703	0.86 [0.50–1.00]	0.039 *
All original_shape_	0.72 [0.50–0.88]	0.131	0.71 [0.50–0.88]	0.048 *	0.64 [0.50–0.88]	0.248	0.51 [0.50–0.62]	0.109	0.68 [0.50–0.97]	0.066
GTV LNM original_	0.60 [0.50–0.70]	0.019 *	0.63 [0.50–0.76]	0.054	0.60 [0.50–0.74]	0.025 *	0.51 [0.50–0.56]	0.012 *	0.57 [0.50–0.69]	0.007 *
GTV LNM original_firstorder_	0.76 [0.51–0.89]	0.032 *	0.81 [0.65–0.96]	0.023 *	0.67 [0.51–0.83]	0.018 *	0.51 [0.50–0.56]	0.006 *	0.60 [0.50–0.76]	0.005 *
GTV LNM original_shape_	0.60 [0.51–0.67]	0.005 *	0.82 [0.56–0.98]	0.005 *	0.72 [0.60–0.81]	0.009 *	0.51 [0.50–0.56]	0.004 *	0.74 [0.62–0.87]	0.003 *
GTV TM original_	0.65 [0.50–0.84]	0.065	0.62 [0.50–0.79]	0.030 *	0.61 [0.51–0.77]	0.007 *	0.59 [0.50–0.74]	0.033 *	0.71 [0.51–0.85]	0.008 *
GTV TM original_firstorder_	0.61 [0.51–0.73]	0.182	0.61 [0.50–0.78]	0.389	0.59 [0.50–0.75]	0.149	0.55 [0.50–0.57]	0.071	0.60 [0.50–0.77]	0.047 *
GTV TM original_shape_	0.61 [0.50–0.73]	0.582	0.60 [0.50–0.80]	0.036 *	0.59 [0.50–0.75]	0.210	0.56 [0.50–0.70]	0.795	0.61 [0.50–0.80]	0.240
Parotid original_	0.68 [0.56–0.76]	0.020 *	0.75 [0.57–0.85]	0.004 *	0.67 [0.59–0.77]	0.001 *	0.54 [0.50–0.60]	0.002 *	0.66 [0.51–0.80]	0.001 *
Parotid original_firstorder_	0.63 [0.52–0.72]	0.006 *	0.68 [0.55–0.81]	0.001 *	0.72 [0.62–0.82]	0.002 *	0.54 [0.50–0.62]	0.019 *	0.66 [0.55–0.79]	0.002 *
Parotid original_shape_	0.57 [0.51–0.74]	0.002 *	0.67 [0.50–0.91]	0.008 *	0.76 [0.55–0.87]	0.001 *	0.52 [0.50–0.58]	0.025 *	0.69 [0.51–0.85]	0.002 *

**Table 3:** Overview of case numbers by dataset.

Dataset	n Training (% Positive)	n Training Data Shapley (% Positive)	n Test (% Positive)	n Selected Features (n All Features)
All original_	29 (66%)	29 (66%)	6 (33%)	2 (321)
All original_firstorder_	29 (66%)	29 (66%)	6 (33%)	2 (60)
All original_shape_	29 (66%)	29 (66%)	6 (33%)	2 (48)
GTV LNM original_	81 (72%)	65 (89%)	15 (40%)	8 (107)
GTV LNM original_firstorder_	81 (72%)	67 (87%)	15 (40%)	8 (20)
GTV LNM original_shape_	81 (72%)	62 (94%)	15 (40%)	8 (16)
GTV TM original_	39 (64%)	39 (64%)	13 (62%)	3 (107)
GTV TM original_firstorder_	39 (64%)	39 (64%)	13 (62%)	3 (20)
GTV TM original_shape_	39 (64%)	39 (64%)	13 (62%)	3 (16)
Parotid original_	78 (67%)	71 (73%)	24 (50%)	7 (107)
Parotid original_firstorder_	78 (67%)	73 (71%)	24 (50%)	7 (20)
Parotid original_shape_	78 (67%)	66 (79%)	24 (50%)	7 (16)

## Data Availability

The datasets generated and/or analyzed during the current study are not publicly available due to the sensitive nature of the research and data safety regulations but are available from the corresponding author upon reasonable request.

## Supplementary Materials

The following supporting information can be downloaded at: https://www.mdpi.com/article/10.3390/cancers15225425/s1. Table S1: Selected features per dataset; Table S2: Descriptive statistics of the patient cohort by HPV status.
