# Supplementary material for: A Radiomics-Based Machine Learning Perspective on the Parotid Gland as a Potential Surrogate Marker for HPV in Oropharyngeal Cancer
Source: Cancers (Basel). 2023 Nov 15;15(22):5425. doi: 10.3390/cancers15225425 (PMC10670647; doi:10.3390/cancers15225425)
Supplement: Supplementary file 1 [file cancers-15-05425-s001.zip › cancers-2625432-supplementary.pdf]

Supplementary Materials

Table S1. Selected features per dataset.

Dataset	Selected Features
All original_	GTV_LNM__original_glcm_Imc1<br>PAROTID__original_shape_Maximum2DDiameterSlice
All original_firstorder_	GTV_LNM__original_firstorder_Minimum<br>PAROTID__original_firstorder_RootMeanSquared
All original_shape_	PAROTID__original_shape_SurfaceVolumeRatio<br>PAROTID__original_shape_Maximum2DDiameterSlice
GTV LNM original_	GTV_LNM__original_firstorder_Median<br>GTV_LNM__original_shape_Elongation<br>GTV_LNM__original_glszm_LargeAreaLowGrayLevelEmphasis<br>GTV_LNM__original_glcm_Contrast<br>GTV_LNM__original_glcm_Imc1<br>GTV_LNM__tech_ConvolutionKernel<br>GTV_LNM__original_glszm_SmallAreaHighGrayLevelEmphasis<br>GTV_LNM__original_firstorder_Minimum
GTV LNM original_firstorder_	GTV_LNM__original_firstorder_Kurtosis<br>GTV_LNM__original_firstorder_Uniformity<br>GTV_LNM__original_firstorder_TotalEnergy<br>GTV_LNM__original_firstorder_Range<br>GTV_LNM__original_firstorder_90Percentile<br>GTV_LNM__original_firstorder_Median<br>GTV_LNM__tech_ConvolutionKernel<br>GTV_LNM__original_firstorder_Minimum
GTV LNM original_shape_	GTV_LNM__original_shape_LeastAxisLength<br>GTV_LNM__original_shape_Maximum2DDiameterColumn<br>GTV_LNM__original_shape_MinorAxisLength<br>GTV_LNM__original_shape_SurfaceVolumeRatio<br>GTV_LNM__original_shape_Sphericity<br>GTV_LNM__original_shape_Flatness<br>GTV_LNM__original_shape_Maximum2DDiameterRow<br>GTV_LNM__tech_ConvolutionKernel
GTV TM original_	GTV_TM__original_glcm_Imc2<br>GTV_TM__original_ngtdm_Complexity<br>GTV_TM__original_glszm_SmallAreaEmphasis
GTV TM original_firstorder_	GTV_TM__original_firstorder_RootMeanSquared<br>GTV_TM__original_firstorder_TotalEnergy<br>GTV_TM__original_firstorder_Range
GTV TM original_shape_	GTV_TM__original_shape_Maximum2DDiameterRow<br>GTV_TM__original_shape_Sphericity<br>GTV_TM__original_shape_Elongation
Parotid original_	PAROTID__original_firstorder_Minimum<br>PAROTID__original_glrlm_LongRunEmphasis<br>PAROTID__original_ngtdm_Coarseness<br>PAROTID__original_glrlm_GrayLevelNonUniformityNormalized<br>PAROTID__original_glrlm_RunLengthNonUniformity<br>PAROTID__original_gldm_DependenceNonUniformity<br>PAROTID__original_firstorder_RootMeanSquared

Parotid original_firstorder_	PAROTID__original_firstorder_Variance<br>PAROTID__original_firstorder_TotalEnergy<br>PAROTID__original_firstorder_InterquartileRange<br>PAROTID__original_firstorder_Uniformity<br>PAROTID__original_firstorder_Kurtosis<br>PAROTID__original_firstorder_Range<br>PAROTID__original_firstorder_RootMeanSquared
Parotid original_shape_	PAROTID__original_shape_MajorAxisLength<br>PAROTID__original_shape_Sphericity<br>PAROTID__original_shape_Flatness<br>PAROTID__original_shape_Maximum3DDiameter<br>PAROTID__original_shape_Maximum2DDiameterSlice<br>PAROTID__original_shape_MeshVolume<br>PAROTID__original_shape_SurfaceArea

Table S2: Descriptive statistics of the patient cohort by HPV status.

	**HPV Negative**	**HPV Positive**
**Patients [n]**	33	20
**Mean age [years] (range)**	58.1 (41–72)	65.2 (45–91)
**Male [%]**	93.9	85.0
**T stage**		
**- T4**	24	11
**- T3**	5	4
**- T1**	2	2
**- T2**	2	3
**N stage**		
**- N0**	3	2
**- N1**	1	4
**- N2**	26	12
**- N3**	3	2
**M stage**		
**- M0**	30	19
**- M1**	2	1
**Grading**		
**- G2**	22	9
**- G3**	10	11
**- G1**	1	0
**Scanner**		
**- No. 1**	13	7
**- No. 2**	12	7
**- No. 3**	8	6
